# Different information needs in subgroups of people with diabetes mellitus: a latent class analysis

**DOI:** 10.1186/s12889-020-09968-9

**Published:** 2020-12-10

**Authors:** Sandra O. Borgmann, Veronika Gontscharuk, Jana Sommer, Michael Laxy, Nicole Ernstmann, Florian M. Karl, Ina-Maria Rückert-Eheberg, Lars Schwettmann, Karl-Heinz Ladwig, Annette Peters, Andrea Icks, for the KORA Study Group

**Affiliations:** 1 Institute for Health Services Research and Health Economics, German Diabetes Center (DDZ), Leibniz Center for Diabetes Research at the Heinrich Heine University Düsseldorf, Auf’m Hennekamp 65, 40225 Düsseldorf, Germany; 2 Institute for Health Services Research and Health Economics, Centre for Health and Society, Faculty of Medicine, Heinrich Heine University Düsseldorf, Düsseldorf, Germany; 3 German Center for Diabetes Research (DZD), Munich-Neuherberg, Germany; 4 Institute of Health Economics and Health Care Management, Helmholtz Zentrum München, German Research Center for Environmental Health, Munich-Neuherberg, Germany; 5 Department of Sport and Health Sciences, Technical University of Munich, Munich, Germany; 6 Center for Health Communication and Health Services Research, Department for Psychosomatic Medicine and Psychotherapy, University Hospital of Bonn, Bonn, Germany; 7 Institute of Epidemiology, Helmholtz Zentrum München, German Research Center for Environmental Health, Munich-Neuherberg, Germany; 8 Chair of Epidemiology, Ludwig-Maximilians-Universität München at UNIKA-T Augsburg, Augsburg, Germany; 9 Department of Economics, Martin Luther University Halle-Wittenberg, Halle an der Saale, Germany; 10 Department of Psychosomatic Medicine and Psychotherapy, Klinikum Rechts der Isar, Technical University of Munich, Munich, Germany

**Keywords:** Patient-centered care, Diabetes mellitus, Needs, Health information

## Abstract

**Background:**

Current evidence suggests that the information needs of people with diabetes mellitus differ across patient groups. With a view to being able to provide individualized information, this study aims to identify (i) the diabetes-related information needs of people with diabetes mellitus; (ii) different subgroups of people with specific information needs; and (iii) associated characteristics of the identified subgroups, such as sociodemographic characteristics, diabetes-related comorbidities, and well-being.

**Methods:**

This cross-sectional study was based on data from 837 respondents with diabetes mellitus who participated in the population-based KORA (Cooperative Health Research in the Augsburg Region) Health Survey 2016 in Southern Germany (KORA GEFU 4 study) (45.6% female, mean age 71.1 years, 92.8% Type 2 diabetes). Diabetes-related information needs were assessed with a questionnaire asking about patients’ information needs concerning 11 diabetes-related topics, e.g. ‘long-term complications’ and ‘treatment/therapy’. Subgroups of people with different information needs and associated characteristics were identified using latent class analysis.

**Results:**

We identified the following four classes of people with different information needs: ‘high needs on all topics’, ‘low needs on all topics’, ‘moderate needs with a focus on complications and diabetes in everyday life’, and ‘advanced needs with a focus on social and legal aspects and diabetes research’. The classes differed significantly in age, years of education, type of diabetes, diabetes duration, diabetes-related comorbidities, smoking behaviour, diabetes education, current level of information, and time preference.

**Conclusions:**

Knowledge about different patient subgroups can be useful for tailored information campaigns or physician-patient interactions. Further research is needed to analyse health care needs in these groups, changes in information needs over the course of the disease, and prospective health outcomes.

**Supplementary Information:**

**Supplementary information** accompanies this paper at 10.1186/s12889-020-09968-9.

## Background

Diabetes mellitus (DM) is a chronic disease with a high prevalence [1, 2], requiring intensive patient self-management [3]. DM is associated with a complex health care situation in which sharing knowledge and providing tailored information that considers patients’ information needs play a key role in patient-centred health care [4]. For example, in physician-patient interactions, knowledge of patients’ information needs can help physicians meet patients’ expectations, leading to informed decision-making and improved health care [5]. However, several barriers have been reported e.g., relevant information was communicated by the physician, but in an unsuitable situation, or the physician did not have enough time to inquire about the patient’s needs [6]. An understanding of what, why, and when information needs arise, is a challenge for health care providers [7]. To support and simplify needs-oriented information exchange, it is of great interest to identify what information is relevant, for what (groups of) patients, and in what stage of the disease.

A few studies have been done on DM regarding patients’ information needs, with initial results indicating differences among patient groups (e.g. age and socioeconomic status) and different phases of the disease (e.g. determined by mode of diabetes treatment and diabetes-related comorbidity) [6, 8–11]. In order to provide individualized information, more work is required to identify information needs-related subgroups in people with DM. In the field of cancer, this question has already been addressed using a specific methodological approach. Neumann and colleagues (2011) used a latent class analysis (LCA) to categorize the information needs of different subgroups of people with cancer and to identify the associated predictors [12]. There is no comparable study among people with DM.

Therefore, the present study aimed to identify (i) the diabetes-related information needs of people with DM; (ii) different subgroups of people with specific information needs; and (iii) associated characteristics of the identified subgroups, such as sociodemographic characteristics, diabetes-related comorbidities and well-being. A patient’s information need is defined as the ‘recognition that their knowledge is inadequate to satisfy a goal, within the context/situation that they find themselves at a specific point in the time’ [7].

## Methods

### Study design and population

This cross-sectional study is based on data from the population-based KORA (Cooperative Health Research in the Augsburg Region) Health Survey 2016 in Southern Germany (KORA GEFU 4 study). KORA has been described in detail elsewhere [13]. KORA is a regional research platform in southern Germany for population-based health surveys. The research platform aims to continue and to expand the project ‘Multinational Monitoring Trends and Determinants in Cardiovascular Disease’ (MONICA) initiated by the World Health Organization in 1984 [14–16]. It examines the links between health, disease, and the living conditions of the population.

In 2016, all 11,189 eligible respondents from the KORA S1–S4 studies (from 1984 to 2001) were invited to participate in the KORA GEFU 4 study (*n* = 9035 responses). An additional diabetes-related questionnaire was sent by post or collected by telephone in 2016 to all eligible Health Survey respondents who reported a diabetes diagnosis (*n* = 1130). The present study included all 837 participants (74.1%) who responded to the diabetes-related questionnaire between May 2016 and January 2017.

### Measurement of diabetes-related information needs

To assess the diabetes-related information needs of individuals with Type 1 and Type 2 diabetes, we used two different sections of the Information Needs in Diabetes Questionnaire (Additional file 1, Appendix 1) [17]:

1. In the first section, respondents were asked to select up to three of 11 diabetes-related topics on which they currently needed more information (multiple answers). This enabled us to identify topics where participants currently had the greatest desire to obtain more information.
2. In the second section, for each of the 11 diabetes-related topics, respondents were asked whether they would like to have more information on each topic at the current time. Response categories were ‘yes’ and ‘no’. Thus, the participants’ information needs were measured for each topic, without being assessed as more or less important, as in the first section.

### Measurement of associated characteristics

Based on the literature (Additional file 2, Appendix 2), we selected characteristics that might be associated with information needs and defined the following six thematic groups of variables:

#### Sociodemographic characteristics [8, 9]

We included age (in years), sex, and years of education (primary education < 11 years vs. secondary/tertiary education ≥11 years).

#### Diabetes-related characteristics [9, 10, 18, 19]

We included type of diabetes, coded as ‘Type 1 diabetes’, ‘Type 2 diabetes’ and ‘other diabetes type’ (e.g. gestational diabetes; not included in the LCA models), and diabetes duration as measured by the question ‘Have you been diagnosed with diabetes by a physician? In which year?’. Antihyperglycaemic medication was coded as ‘yes’ if respondents stated that they currently took oral glucose-lowering drugs or insulin. If both treatment options were answered with ‘no’, the variable was coded as ‘no’. Diabetes-related comorbidities were coded as ‘yes’ if respondents reported at least one of the following comorbidities (retinopathy, blindness, microalbuminuria, kidney failure, artificial kidney, peripheral artery occlusive disease, polyneuropathy, diabetic foot syndrome, amputation). Otherwise, no comorbidity was assumed and the characteristic coded as ‘no’.

#### Lifestyle-related characteristic [9, 20]

Smoking behaviour was assessed in terms of the current smoking situation, with the responses ‘yes’ (regularly or occasionally) or ‘no’ (never- or ex-smoker).

#### Well-being [10, 21]

Well-being was measured with the German version of the World Health Organisation-Five Well-Being Index (WHO-5) [22] and coded as ‘low well-being’ or ‘high well-being’ (cut-off score ≥ 50).

#### Current level of information and diabetes education [10]

The respondents’ current level of diabetes-related information was captured using the Information Needs in Diabetes Questionnaire [17]. Responses for the 11 topics (Additional file 1, Appendix 1) were given on a 4-point Likert scale from ‘not informed at all’, coded as 0, to ‘very well informed’, coded as 3. The sum of all variables ranged from 0 to 33. In addition, we included participation in a diabetes training programme (yes vs. no) as a measure of diabetes education.

#### Time preference [9, 23]

To measure time preference, we asked respondents to indicate their level of agreement to the statement: ‘My present well-being is more important to me than my future health status’ on a 4-point Likert scale which was then dichotomised and coded as ‘rather disagree’ or ‘rather agree’. Time preference can be regarded as present orientation as it assessed ‘whether participants preferred immediate pleasure over long-term health’ [24].

### Statistical analysis

The descriptive analysis included frequencies, mean values and standard deviations. All quantitative analyses were performed in the SAS software, V.9.4 (SAS Institute Inc., Cary, NC).

#### Analysis of information needs using LCA: handling of missing data

In total, 708 participants (84.6%) responded to at least one topic of the Information Needs in Diabetes Questionnaire (Section 1 or Section 2, see ‘Measurement of diabetes-related information needs’). The LCA [25] was performed with the data from the second section. Four hundred eighty participants (57.3%) provided information for at least one of the 11 topics in Section 2 (a question asking whether the participant needs information on each topic, with yes/no as the possible responses), while 283 respondents (33.8%) provided information for all 11 topics.

The missing values seemed to be not at random: Frequency of answering ‘yes’ (reporting an information need) increased for all 11 questions as the total number of missing values increased (Additional file 2, Appendix 3). Therefore, it seemed that some respondents understood this section more as a checklist in which it was not necessary to select the answer ‘no’; one only needed to answer a question with ‘yes’ if information on a given topic was needed. Hence, missing values can be assumed to at least partially reflect the answer ‘no’ (‘checklist misconception effect’ [26]).

Therefore, we decided to perform a main analysis and two sensitivity analyses in which the missing values were handled differently. In the main analysis (Variant 1), we did not perform any imputation at all. We included only respondents who answered at least one of the 11 questions of Section 2 of the questionnaire (*n* = 480) and used the answers that were available. In the first sensitivity analysis (Variant 2), all missing values for the 11 questions of Section 2 were coded with ‘no’ and the LCA was performed with the full sample (*n* = 837). In the second sensitivity analysis (Variant 3), missing values were coded as ‘yes’ if the respective diabetes-related topic was selected as one of the three most important topics in the first section of the Information Needs in Diabetes Questionnaire. We assumed that participants who had already reported a need for information in the first part of the questionnaire might not want to mention it again. This led to a total of 613 participants in Variant 3.

#### LCA without covariates: identification of subgroups with different information needs

In line with Lanza and colleagues (2007), we calculated LCA models without covariates with one to eight classes in order to identify the optimal number of classes [25]. We chose the best model based on model fit indicators. Lower values of the Bayesian information criterion (BIC) and adjusted Bayesian information criterion (aBIC) indicated better fit. A (relative) entropy close to one indicated high separation of classes. Moreover, we assessed whether the classes were meaningful, set the minimum class prevalence to 5%, and took the ‘law of parsimony’ [27] into account.

#### LCA with covariates: identification of associated characteristics

After selecting the number of classes, we performed LCA with covariates to investigate the characteristics associated with the identified subgroups. LCA with the following covariates were performed: age, sex, years of education, type of diabetes, diabetes duration, antihyperglycaemic medication, diabetes-related comorbidities, current smoking behaviour, well-being, diabetes education, current level of information, and time preferences (see ‘Measurement of associated characteristics’).

## Results

### Description of population

#### Respondent characteristics

The sample (*n* = 837) is described in detail in Table 1.

**Table 1:** Participants’ characteristics

Characteristics	n (%) / (M ± SD)
Total number of participants	837
Age (years), *n* = 837	71.1 ± 9.5
Sex, *n* = 837	
*Female*	382 (45.6)
*Male*	455 (54.4)
Years of education, *n* = 836	
*≥ 11 years*	330 (39.5)
*< 11 years*	506 (60.5)
Type of diabetes, *n* = 804	
*Type 1 diabetes*	41 (5.1)
*Type 2 diabetes*	746 (92.8)
*Other diabetes type*	17 (2.1)
Diabetes duration, *n* = 763	
*≥ 10 years*	394 (51.6)
*< 10 years*	369 (48.4)
	12.1 ± 10.2
Mode of diabetes treatment, *n* = 835	
*No oral glucose-lowering drugs and no insulin*	145 (17.4)
*Oral glucose-lowering drugs*	482 (57.7)
*Insulin*	99 (11.9)
*Oral glucose-lowering drugs and insulin*	109 (13.1)
Diabetes-related comorbidity, *n* = 837	
*Yes*	329 (39.3)
*No*	508 (60.7)
Current smoking behaviour, *n* = 834	
*Regular*	89 (10.7)
*Occasionally*	8 (1.0)
*Ex-smoker*	380 (45.6)
*Never*	357 (42.8)
Well-being, *n* = 834	
*High well-being (≥50)*	596 (71.5)
*Low well-being (< 50)*	238 (28.5)
Current level of information, *n* = 505	16.2 ± 6.8
Diabetes education, *n* = 731	
*Yes*	386 (52.8)
*No*	345 (47.2)
Time preference, *n* = 697	
*Rather agree*	296 (42.5)
*Rather disagree*	401 (57.5)

*M* mean, *SD* standard deviation

Mode of diabetes treatment: Self-reported treatment. Diabetes-related comorbidity: Included were retinopathy, blindness, microalbuminuria, kidney failure, artificial kidney, peripheral artery occlusive disease, polyneuropathy, diabetic foot syndrome, and amputation. Well-being: High well-being was reported if WHO-5 Index ≥50 [22]. Current level of information: Responses for eleven diabetes-related topics were given on a 4-point Likert scale. The sum of all variables ranged from 0 to 33 (higher scores indicate a higher level of information). Diabetes education: Answer to the question of whether the participant has participated in a diabetes training program. Time preference: Agreement with the statement ‘My present well-being is more important to me than my future health status’

#### Diabetes-related information needs

**Fig. 1:**
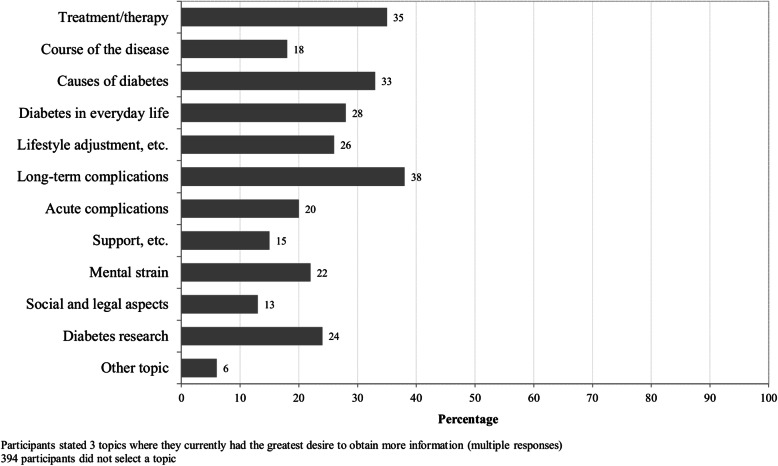
Topics where respondents currently had the greatest desire to obtain more information (respondents who provided at least one answer in the first part of the Information Needs in Diabetes Questionnaire, *n* = 443 (52.9% of the study population))

In the first part of the Information Needs in Diabetes Questionnaire, 443 respondents (52.9%) selected one to three diabetes-related topics they currently considered as most important in terms of a need for more information (Fig. 1). Most respondents selected information regarding ‘long-term complications’ (37.7%) and ‘treatment/therapy’ (35.0%) as most important. The topics ‘support, helplines, and information sources’ (14.7%) and ‘social and legal aspects’ (12.6%) were less frequently chosen.

In the second part of the Information Needs in Diabetes Questionnaire, 480 respondents (57.3%) indicated whether they had an information need for at least one of the 11 topics, as already mentioned in the method section (Additional file 2, Appendix 4). For each topic, 28–42% of the respondents indicated that they would like to have more information at the current time. The percentage was highest for ‘diabetes research’ (41.9%) and ‘long-term complications’ (39.7%), and lowest for ‘support, helplines, and information sources’ (27.9%).

### Subgroups with specific information needs

#### LCA without covariates: Identification of subgroups with different information needs

In the main analysis and the two sensitivity analyses, the four-class model exhibited the best fit. The four-class model had the lowest aBIC value across all three variants of LCA and a high entropy score. The model fit indicators are described in detail in Additional file 2, Appendix 5. In addition, the four-class model was the most substantially meaningful. Therefore, in line with the LCA literature [25], we selected the model with four classes as most suitable.

**Fig. 2:**
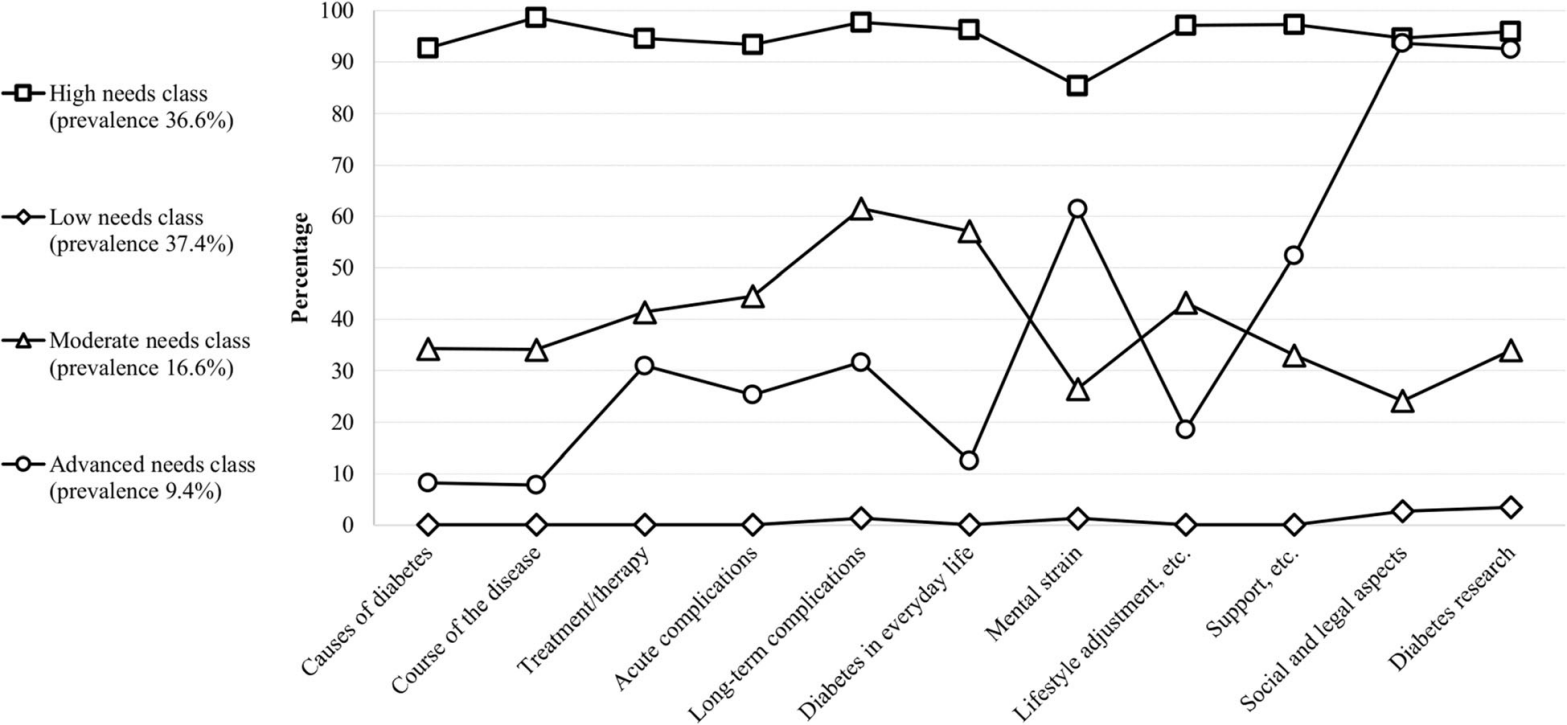
Probabilities of information needs stratified by LCA classes in the main analysis (Variant 1) without covariates (*n* = 480)

The four identified classes represented four different information needs profiles in people with DM. They differed considerably in nearly all of the 11 diabetes-related topics and can be described as follows (Fig. 2):

- *High information needs on all topics (high needs class):* The probability of a reported information need was 85.3 to 98.7% for all topics. The estimated class prevalence was 36.6%.
- *Low information needs on all topics (low needs class):* The probability of a reported information need was 0.0 to 3.5% for all topics. The estimated class prevalence was 37.4%.
- *Moderate information needs with a focus on complications and diabetes in everyday life (moderate needs class):* The probability of a reported information need was highest for ‘long-term complications’ (61.6%), ‘diabetes in everyday life’ (57.1%), ‘acute complications’ (44.5%) and ‘lifestyle adjustment, health promotion and prevention’ (43.2%). For other topics, the probability of a reported information need was between 24.1 and 41.4%. The estimated class prevalence was 16.6%.
- *Advanced information needs with a focus on social and legal aspects and diabetes research (advanced needs class):* The probability of a reported information need for ‘social and legal aspects’ and ‘diabetes research’ was 93.7 and 92.5%, respectively. For the topics ‘mental strain’ and ‘support, helplines, and information sources’, the probability of a reported information need was 61.5 and 52.3%, respectively. For the seven other topics, the probability of a reported information need was between 8.2 and 31.6%. The estimated class prevalence was 9.4%.

In the sensitivity analyses, the classes were very comparable (Additional file 2, Appendix 6). However, the prevalence of the classes differed. In particular, the prevalence of the ‘high needs class’ and the ‘low needs class’ changed due to the different handling of missing values.

#### LCA with covariates: identification of associated factors

The variables age, years of education, type of diabetes, diabetes duration, diabetes-related comorbidities, smoking behaviour, diabetes education, current level of information, and time preference differed significantly among the four classes of information needs (Table 2).

**Table 2:** LCA main analysis (Variant 1) with covariates (*n* = 306)

	Low needs vs. high needs		Moderate needs vs. high needs		Advanced needs vs. high needs		Moderate needs vs. low needs		Advanced needs vs. low needs		Advanced needs vs. moderate needs	
	OR	CI 95%	OR	CI 95%	OR	CI 95%	OR	CI 95%	OR	CI 95%	OR	CI 95%
Age (years)	**1.06**	**[1.03; 1.09]**	**1.04**	**[1.01; 1.07]**	1.01	[0.98; 1.05]	0.98	[0.95; 1.01]	**0.95**	**[0.92; 0.99]**	0.97	[0.94; 1.01]
Sex (female)	1.19	[0.74; 1.91]	1.22	[0.71; 2.09]	1.15	[0.61; 2.17]	1.03	[0.62; 1.71]	0.97	[0.54; 1.75]	0.94	[0.49; 1.83]
Years of education (≥11 years)	1.01	[0.63; 1.61]	1.47	[0.87; 2.48]	**2.21**	**[1.20; 4.09]**	1.46	[0.89; 2.39]	**2.19**	**[1.23; 3.91]**	1.50	[0.79; 2.86]
Type of diabetes (Type 2)	**0.17**	**[0.05; 0.56]**	0.74	[0.12; 4.66]	0.34	[0.08; 1.38]	4.34	[0.89; 21.17]	1.98	[0.67; 5.83]	0.45	[0.07; 2.98]
Diabetes duration (years)	**0.97**	**[0.95; 0.99]**	**0.97**	**[0.94; 0.998]**	1.00	[0.97; 1.03]	1.00	[0.97; 1.03]	**1.03**	**[1.01; 1.06]**	1.03	[0.995; 1.07]
Antihyperglycaemic medication (yes)	0.88	[0.44; 1.76]	0.86	[0.40; 1.85]	0.77	[0.30; 1.99]	0.98	[0.50; 1.90]	0.87	[0.37; 2.08]	0.89	[0.35; 2.26]
Comorbidities (yes)	**0.44**	**[0.27; 0.73]**	**0.55**	**[0.31; 0.98]**	0.83	[0.43; 1.57]	1.25	[0.73; 2.12]	**1.86**	**[1.03; 3.38]**	1.49	[0.77; 2.92]
Current smoking behaviour (yes)	0.98	[0.51; 1.89]	0.44	[0.18; 1.06]	**0.12**	**[0.03; 0.50]**	0.44	[0.19; 1.04]	**0.12**	**[0.03; 0.49]**	0.28	[0.06; 1.27]
High well-being (≥50)	1.21	[0.71; 2.08]	0.85	[0.47; 1.56]	0.67	[0.34; 1.34]	0.70	[0.39; 1.26]	0.56	[0.29; 1.07]	0.79	[0.38; 1.63]
Diabetes education (yes)	1.13	[0.70; 1.82]	1.15	[0.67; 1.98]	**1.98**	**[1.03; 3.82]**	1.02	[0.62; 1.69]	1.75	[0.94; 3.27]	1.72	[0.87; 3.39]
Current level of information	**1.14**	**[1.09; 1.18]**	1.01	[0.97; 1.05]	**1.11**	**[1.05; 1.17]**	**0.89**	**[0.85; 0.92]**	0.98	[0.93; 1.03]	**1.10**	**[1.04; 1.16]**
Time preference (rather agree)	**2.05**	**[1.24; 3.38]**	1.18	[0.66; 2.09]	0.60	[0.28; 1.27]	**0.58**	**[0.34; 0.97]**	**0.29**	**[0.14; 0.59]**	0.51	[0.24; 1.09]

*OR* odds ratio (corresponding to one unit change in age, diabetes duration and current level of information)

*CI* confidence interval

**significant results (*p*** **< 0.05)**

The present findings suggest that the typical person with ‘*high needs*’ is younger than a person with ‘*low needs*’ and ‘*moderate needs*’, less educated than a person with ‘*advanced needs*’ and is more likely to have Type 2 diabetes than a person with ‘*low needs*’. They have a longer duration of diabetes and is more likely to have comorbidities than a person with ‘*low needs*’ or *‘moderate needs*’. In addition, the person is more likely to smoke and less likely to have participated in a diabetes education program than a person with ‘*advanced needs*’. A person with ‘*high needs*’ is also less informed than a person with ‘*low needs*’ or ‘*advanced needs*’ and is more likely to disagree that current well-being is more important than future health than a person with ‘*low needs*’.

A typical person with ‘*low needs*’ is older than a person with ‘*high needs*’ or ‘*advanced needs*’, less educated than a person with ‘*advanced needs*’ and more likely to have Type 1 diabetes than a person with ‘*high needs*’. They have a shorter duration of diabetes and are less likely to have comorbidities than a person with ‘*high needs*’ or ‘*advanced needs*’. In addition, the person is more likely to smoke than a person with ‘*advanced needs*’. A person with ‘*low needs*’ is also better informed than a person with ‘*high needs*’ or ‘*moderate needs*’ and more likely to agree that current well-being is more important than future health than persons with the other three needs profiles.

A typical person with ‘*moderate needs with a focus on complications and diabetes in everyday life’* is older, has a shorter duration of diabetes and less likely to have comorbidities than a person with ‘*high needs*’. They are less informed than a person with ‘*low needs*’ or ‘*advanced needs*’ and more likely to disagree that current well-being is more important than future health than persons with ‘*low needs*’.

A typical person with ‘*advanced needs with a focus on social and legal aspects and diabetes research’* is younger than a person with ‘*low needs*’, better educated than a person with ‘*high needs*’ or ‘*low needs*’, and has a longer duration of diabetes and is more likely to have comorbidities than a person with ‘*low needs*’. In addition, they are less likely to smoke than a person with ‘*high needs*’ or ‘*low needs*’, more likely to have participated in a diabetes education program than a person with ‘*high needs*’, and better informed than a person with ‘*high needs*’ or ‘*moderate needs*’. The person is more likely to disagree that current well-being is more important than future health than a person with ‘*low needs*’.

The covariates ‘sex’, ‘antihyperglycaemic medication’, and ‘well-being’ exhibited no significant associations (Additional file 2, Appendix 7 for Variant 2 and Variant 3). Respondents’ characteristics stratified by classes are presented in Table 3 (Additional file 2, Appendix 8 for Variant 2 and Variant 3) and Table 4.

**Table 3:** Participants’ characteristics stratified by classes (main analysis with covariates, *n* = 306)

Characteristics	High needs class n (%) / (M ± SD)	Low needs class n (%) / (M ± SD)	Moderate needs class n (%) / (M ± SD)	Advanced needs class n (%) / (M ± SD)
**N**	85	120	60	41
**Age (years)**	67.1 ± 9.1	70.3 ± 8.0	70.3 ± 9.5	65.9 ± 10.4
**Sex (female)**	32 (37.6)	55 (45.8)	25 (41.7)	18 (43.9)
**Years of education (≥11 years)**	34 (40.0)	46 (38.3)	30 (50.0)	26 (63.4)
**Type of diabetes (Type 2)**	83 (97.6)	109 (90.8)	60 (100.0)	35 (85.4)
**Diabetes duration (years)**	13.0 ± 10.5	11.9 ± 8.9	9.6 ± 9.6	14.9 ± 12.5
**Antihyperglycaemic medication (yes)**	74 (87.1)	101 (84.2)	49 (81.7)	37 (90.2)
**Comorbidities (yes)**	42 (49.4)	40 (33.3)	22 (36.7)	24 (58.5)
**Current smoking behaviour (yes)**	15 (17.6)	21 (17.5)	4 (6.7)	0 (0.0)
**High well-being (≥50)**	59 (69.4)	93 (77.5)	43 (71.7)	28 (68.3)
**Diabetes education (yes)**	44 (51.8)	68 (56.7)	27 (45.0)	32 (78.0)
**Current level of information**	13.0 ± 6.0	18.9 ± 7.0	11.8 ± 5.5	18.3 ± 4.3
**Time preference (rather agree)**	23 (27.1)	60 (50.0)	21 (35.0)	5 (12.2)

**Table 4:** Description of class characteristics compared to the other classes (main analysis with covariates, *n* = 306)

Selected characteristics	Typical person with …			
	High needs: High information needs on all topics	Low needs: Low information needs on all topics	Moderate needs: Moderate information needs with a focus on complications and diabetes in everyday life	Advanced needs: Advanced information needs with a focus on social and legal aspects and diabetes research
**Age**	*Younger*	*Older*	*Older*	*Younger*
**Years of education**	*Lower educated*	*Lower educated*	Better educated	*Better educated*
**Type of diabetes**	*Type 2*	*Type 1 and Type 2*	Type 2	Type 1 and Type 2
**Diabetes duration**	*Longer duration*	*Shorter duration*	*Shorter duration*	*Longer duration*
** Comorbidities**	*Yes*	*No*	*No*	*Yes*
**Current smoking behaviour**	*Yes*	*Yes*	No	*No*
**Diabetes education**	*No*	Yes	No	*Yes*
**Current level of information**	*Lower level of information*	*Higher level of information*	*Lower level of information*	*Higher level of information*
**Time preference**	*Rather disagree*	*Rather agree*	*Rather disagree*	*Rather disagree*

The results were extracted from the LCA main analysis (Table 2 and Table 3)

Significant results (Table 2) are *marked in italics*

## Discussion

### Main findings

We identified a number of individuals with DM with diabetes-related information needs. The following four classes of people with different information needs were found: ‘*high needs on all topics*’; ‘*low needs on all topics*’; ‘*moderate needs with a focus on complications and diabetes in everyday life*’ and ‘*advanced needs with a focus on social and legal aspects and diabetes research*’. The classes differed significantly in age, years of education, type of diabetes, diabetes duration, diabetes-related comorbidities, smoking behaviour, diabetes education, current level of information, and time preference.

### Discussion of the findings and comparison to other studies

This is the first study to identify subgroups of persons with DM with different sets of information needs. Recent studies have analysed information needs among people with DM more generally, with some finding higher information needs than in our study [8–10, 28]. One reason for this observation might be differences in the study samples. For example, only participants with a diabetes duration of less than 1 year are included in the German Diabetes Study [10]. More than 57% of the participants stated that they had a need for information on each diabetes-related topic [10]. It has been suggested that information needs may change over the course of the disease [6, 19, 29, 30]. It is therefore conceivable that individuals with a recent diagnosis represent a subgroup with specific (higher) information needs.

We identified a class with *‘low needs’*. Compared to the other classes, these respondents felt well informed. In addition to respondents who are already well informed, this subgroup may also include respondents who are not aware of their lack of knowledge [6]. According to a systematic review by Horigan et al. (2017), people with DM’s perceived level of information can account for non-participation in diabetes training programmes [31]. Individuals felt that they had already received enough information about diabetes [31]. Other authors also reported problems related to health information overload, especially among people with low education and low health literacy [5, 32, 33]. Furthermore, individuals with *‘low needs’* were more likely to be present-biased than future-biased (i.e. preferring smaller, more immediate rewards to larger, later rewards). They tended to agree that their current well-being is more important to them than their future health state. In contrast, respondents with ‘*high needs*’ or ‘*advanced needs*’ were more future-biased. A previous systematic review showed that present-biased individuals with DM had worse diabetes self-care and worse HbA_1c_ values [34]. Further studies should be carried out to analyse possible associations with information needs.

We identified a second class encompassing individuals with ‘*high needs*’ for information on all diabetes-related topics with no thematic focus. Crangle and colleagues (2018) reported a change in the need for information upon the onset of complications, noting that ‘with the onset of complications, a person may be energized by the complications to manage his/her diabetes’ [30]. Ormandy (2011) also reported that the perception of disease-related symptoms drives the recognition and verbalization of information needs [7]. In line with these results, we found that respondents with *‘high needs’* were more likely to have comorbidities than respondents with *‘low needs’* and *‘moderate needs’*.

We found that respondents with ‘*moderate needs*’ had a more thematic focus than respondents with ‘*high needs*’ or ‘*low needs*’. This thematic focus includes complications and daily life, which are also described as important in other studies on diabetes-related information needs [9]. Given these prevention-related topics, the moderate needs and the fact that the group tends to have fewer comorbidities, it may be worthwhile to pay attention to this class and its information seeking behaviour including potential motivating (e.g. fear of future physical state) and demotivating factors (e.g. stigma) [6].

In the *‘advanced needs’* class, respondents wished more information regarding ‘social and legal aspects’ and ‘diabetes research’. We assume that they lack information that is less often provided in medical treatment. In an earlier study, we also found that perceived current level of information on the topics ‘social and legal aspects’ and ‘diabetes research’ was lower compared to the other topics [9, 10]. This indicates that there is an information deficit at the system level.

### Implications

We identified a number of individuals with DM who exhibited diabetes-related information needs. Therefore, efforts should be extended in this area, and the provision of information should be developed to better meet patients’ needs. For example, knowledge about the four different subgroups and their associated characteristics can be used in tailored information campaigns, physician-patient interactions or in online health information websites. Here, some specific aspects could be taken into account: (1) Following Madsen and colleagues (2019), it is conceivable that present-biased people with ‘*low needs*’ will probably not benefit much from information campaigns. It seems that offers intended to improve outcomes in line with persons’ actual interests and to reduce self-care costs are more suitable. (2) It may be meaningful to provide general access to a wide range of information, but to personalise it according to each patient’s needs. More attention must be paid to information that is more difficult to obtain and goes beyond the medical context. Researchers should also see themselves as obliged to disseminate their results to target groups beyond the scientific community. (3) In providing information in accordance with peoples’ needs, the factors ‘current level of information’, ‘time preferences’ and ‘comorbidities’ may turn out to be good indicators for current information needs. Considering these factors in information transfer, e.g. by using a questionnaire in physician-patient communication or inquiring about this information on websites, could contribute to this improvement.

### Limitations and strengths

The main limitation of this study is the high number of missing values, especially in the information needs variables. Class prevalence should be interpreted with caution. In the main analysis, no imputation was performed. Since it is likely that missing values reflect ‘no information need’, the prevalence of the ‘*high needs class*’ in particular may be overestimated. However, in light of the stable results in the sensitivity analyses, we assume that the identified classes and associated factors are not biased by missing values. Another limitation of our study is its cross-sectional design. It would be interesting to determine whether allocation to one of the four identified classes of information needs can predict a person’s health outcomes. In addition, the sample may be biased due to non-responses. It was shown that non-responders in KORA studies were older and less healthy [35–37]. We assume that this also applies to the population in our study. The use of data generated through self-reporting is another limitation, especially with regard to some associated factors as for example current smoking behaviour. These results should be interpreted with caution. However, self-reporting appears to be a successful method of assessing the views of people with DM, for example to evaluate their individual needs.

An important strength of this study is the population-based study design. Another strength is that the LCA classes differed from each other with respect to several variables, and the identified classes and associations with other characteristics were stable across all LCA variants. Due to these stable results, it is possible to identify specific implications for the individual classes.

## Conclusion

Although people with DM are unique and the population of people with DM is heterogeneous, four different subgroups with respect to diabetes-related information needs were identified. These subgroups differed in demographic, socioeconomic, diabetes-related and other personal characteristics (e.g. time preferences). While from a clinician’s perspective it could be argued that each client’s information needs should be individually assessed and the information provided tailored to meet their requirements, understanding the information needs of subgroups of people with DM is beneficial when considering the development of targeted resources such as education programs, written educational material, websites and peer support models. Future work should investigate the health care needs of these groups, changes in information needs over the course of the disease, possible associations with coping strategies, and prospective health outcomes.

## Supplementary Information

**Additional file 1: Appendix 1.** Information Needs in Diabetes Questionnaire

**Additional file 2: Appendix 2.** Rationale of the variables used in the LCA (association with information needs). **Appendix 3.** Relative frequency of current information needs stratified by groups of people with different numbers of missing values in the second part of the Information Needs in Diabetes Questionnaire. **Appendix 4.** Information needs of study participants**. Appendix 5.** Statistical parameters for models with different numbers of classes per LCA variant**. Appendix 6.** Probabilities of information needs among people with diabetes stratified by the identified LCA classes in the sensitivity analyses without covariates. **Appendix 7.** LCA with covariates per LCA variant. **Appendix 8.** Participants’ characteristics stratified by classes per LCA variant with covariates

## Data Availability

Data may be obtained from a third party and are not publicly available. Project agreements to use and access KORA data can be requested from national and international researchers via the KORA-PASST tool under https://epi.helmholtz-muenchen.de/.

## Abbreviations

*aBIC*: Adjusted Bayesian information criterion
*BIC*: Bayesian information criterion
*DM*: Diabetes mellitus
*KORA GEFU 4 study*: Cooperative Health Research in the Augsburg Region Health Survey
*LCA*: Latent class analysis
*WHO-5*: World Health Organization-Five Well-Being Index
