# Supplementary material for: Different information needs in subgroups of people with diabetes mellitus: a latent class analysis
Source: BMC Public Health. 2020 Dec 10;20:1901. doi: 10.1186/s12889-020-09968-9 (PMC7730786; doi:10.1186/s12889-020-09968-9)
Supplement: Supplementary file 1 — Additional file 1: Appendix 1. Information Needs in Diabetes Questionnaire [file 12889_2020_9968_MOESM1_ESM.pdf]

Appendix 1

Information Needs in Diabetes Questionnaire

1. Listed below are various topics relating to diabetes. Please have a look at this list and then consider which three topics you would currently like to have further information about. Finally, please enter these topics in the answer boxes below and explain what particular interests you have about these topics.

**Topics relating to diabetes**	
A	Causes of diabetes
B	Course of the disease
C	Treatment/therapy
D	Acute complications
E	Late complications
F	Diabetes in everyday life
G	Mental strain
H	Lifestyle adjustment, health promotion and prevention
I	Support, helplines and information sources
J	Social and legal aspects
K	Scientific surveys and research on diabetes
L	Other topics not included in the list

Please enter the letters representing the three topics about which you would currently like to have further information. Please explain what particularly interests you have about these topics.

**Topic**	**I am particularly interested in**
☐	____________________
	____________________
☐	____________________
	____________________
☐	____________________
	____________________

2. Please specify how well informed you are on the following topics and whether you currently wish to have further information on each of these topics.

**How well informed are you on the following topics?**					**Would you currently like information on the topic?**	
Causes of diabetes	☐ very well	☐ well	☐ not well	☐ not informed at all	☐ yes	☐ no
Course of the disease	☐ very well	☐ well	☐ not well	☐ not informed at all	☐ yes	☐ no
Treatment/therapy	☐ very well	☐ well	☐ not well	☐ not informed at all	☐ yes	☐ no
Acute complications	☐ very well	☐ well	☐ not well	☐ not informed at all	☐ yes	☐ no
Late complications	☐ very well	☐ well	☐ not well	☐ not informed at all	☐ yes	☐ no
Diabetes in everyday life	☐ very well	☐ well	☐ not well	☐ not informed at all	☐ yes	☐ no
Mental strain	☐ very well	☐ well	☐ not well	☐ not informed at all	☐ yes	☐ no
Lifestyle adjustment, health promotion and prevention	☐ very well	☐ well	☐ not well	☐ not informed at all	☐ yes	☐ no
Support, helplines and information sources	☐ very well	☐ well	☐ not well	☐ not informed at all	☐ yes	☐ no
Social and legal aspects	☐ very well	☐ well	☐ not well	☐ not informed at all	☐ yes	☐ no

How well informed are you on the following topics?					Would you currently like information on the topic?	
Scientific surveys and research on diabetes	☐ very well	☐ well	☐ not well	☐ not informed at all	☐ yes	☐ no
Other topics not included in the list: ________________________	☐ very well	☐ well	☐ not well	☐ not informed at all	☐ yes	☐ no

3. What do you consider to be particularly important with regard to information on diabetes?

____

____

____

____

____
