# Supplementary material for: Different information needs in subgroups of people with diabetes mellitus: a latent class analysis
Source: BMC Public Health. 2020 Dec 10;20:1901. doi: 10.1186/s12889-020-09968-9 (PMC7730786; doi:10.1186/s12889-020-09968-9)
Supplement: Supplementary file 2 — Additional file 2: Appendix 2. Rationale of the variables used in the LCA (association with information needs). Appendix 3. Relative frequency of current information needs stratified by groups of people with different numbers of missing values in the second part of the Information Needs in Diabetes Questionnaire. Appendix 4. Information needs of study participants. Appendix 5. Statistical parameters for models with different numbers of classes per LCA variant. Appendix 6. Probabilities of information needs among people with diabetes stratified by the identified LCA classes in the sensitivity analyses without covariates. Appendix 7. LCA with covariates per LCA variant. Appendix 8. Participants’ characteristics stratified by classes per LCA variant with covariates [file 12889_2020_9968_MOESM2_ESM.pdf]

# Appendix 2

## Rationale of the variables used in the LCA (association with information needs)

| Variable group | Rationale |
|---|---|
| **(i) Sociodemographic characteristics** | Sociodemographic factors were considered less in studies on information needs among people with DM [9]. Duggan and Bates (2008) showed that the need for medical information decreased with age. Moreover, a higher socioeconomic status (defined by occupation) was associated with a higher need for drug information [8]. |
| **(ii) Diabetes-related characteristics** | Recent studies have addressed the information needs of people with Type 2 and Type 1 diabetes, but little attention has been paid to comparing them [9]). Some studies reported changes in information needs over the course of the disease [9, 18, 19]. Antihyperglycaemic medication was associated with higher information needs and comorbidities were associated with lower information needs in people with recently diagnosed Type 2 diabetes [10]. |
| **(iii) Lifestyle-related characteristics** | People with DM have shown to be highly interested in lifestyle-related information [9, 20], so we assume an association with lifestyle-related characteristics. |
| **(iv) Well-being** | Improving health-related quality of life is a goal of patient-centred collaborative diabetes care [21]. In people with Type 1 diabetes, a higher mental component summary score on the 36-item Short-Form Health Survey was associated with a lower information need on management-related topics (e.g. diabetes in everyday life) [10]. |
| **(v) Current level of information and diabetes education** | Current level of information regarding diabetes-related topics has been shown to be associated with information needs [10]. |
| **(vi) Time preference** | A systematic review showed that present-biased individuals with diabetes had worse diabetes self-care and worse $HbA_{1c}$ values [34]. Therefore, we assume that there might also be an association with information needs. |

# Appendix 3

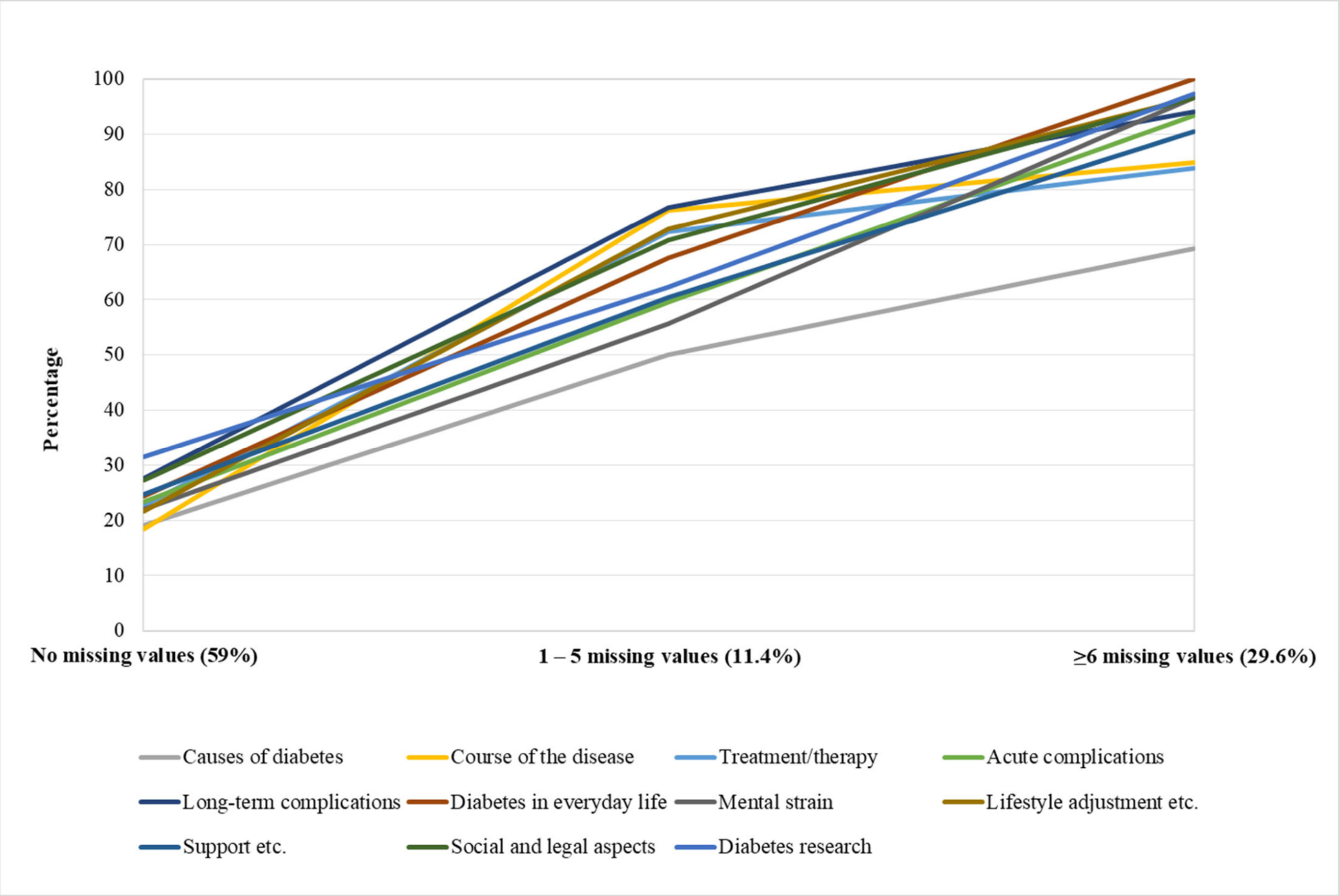


**Relative frequency of current information needs stratified by groups of people with different numbers of missing values (n=480) in the second part of the Information Needs in Diabetes Questionnaire** (Frequency of answering 'yes' among three groups of people with different numbers of missing values on the 11 items. People with six or more missing values for the 11 items were more likely to answer 'yes' to the remaining items than people with fewer missing values.)

# Appendix 4

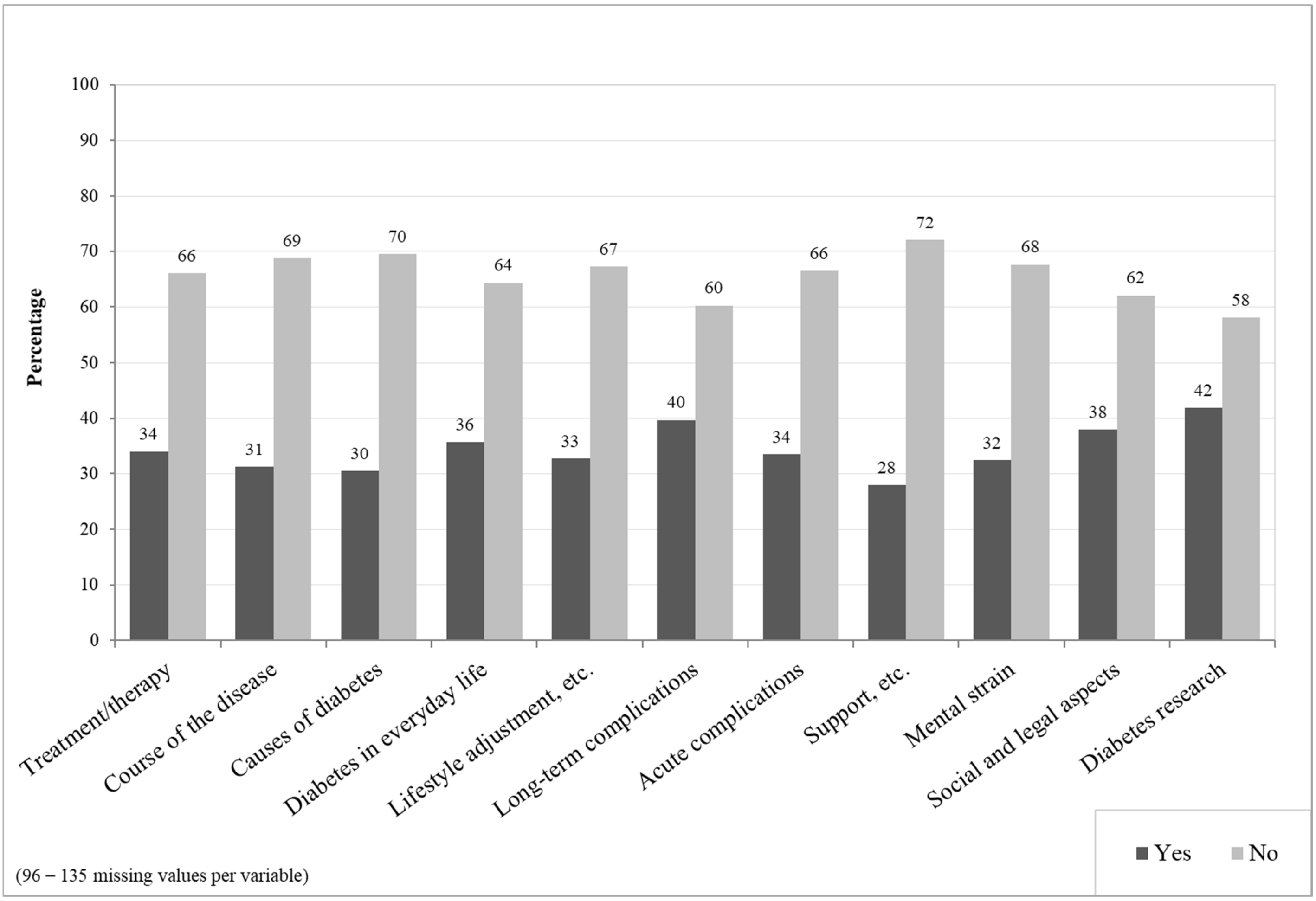


**Information needs of study participants** (respondents with at least one answer in the second part of the Information Needs in Diabetes Questionnaire, n=480 (57.3% of the study population))

# Appendix 5

*Statistical parameters for models with different numbers of classes per LCA variant*

| Number of classes | | BIC | CAIC | aBIC | ENTROPY |
|---|---|---|---|---|---|
| **1** | | | | | |
| | *Variant 1* | 2796.0346037 | 2807.0346037 | 2761.1217496 | 1 |
| | *Variant 2* | 3581.6376683 | 3592.6376683 | 3546.7053291 | 1 |
| | *Variant 3* | 3415.2105968 | 3426.2105968 | 3380.2878353 | 1 |
| **2** | | | | | |
| | *Variant 1* | 1174.5944106 | 1197.5944106 | 1101.5948067 | 0.9020362897 |
| | *Variant 2* | 1465.6858558 | 1488.6858558 | 1392.6455104 | 0.9625185671 |
| | *Variant 3* | 1244.1433757 | 1267.1433757 | 1171.1230562 | 0.9329189586 |
| **3**[a] | | | | | |
| | *Variant 1* | **814.6334849** | **849.6334849** | 703.54713119 | 0.8774056165 |
| | *Variant 2* | 1216.0600868 | 1251.0600868 | 1104.911735 | 0.9428111275 |
| | *Variant 3* | **832.9436729** | **867.9436729** | 721.8257954 | 0.8964830286 |
| **4** | | | | | |
| | *Variant 1* | 821.45607569 | 868.45607569 | **672.28297214** | 0.8603133289 |
| | *Variant 2* | **1166.323633** | **1213.323633** | **1017.0672749** | 0.9273188098 |
| | *Variant 3* | 840.34122623 | 887.34122623 | **691.1257908** | 0.8838814602 |
| **5**[b] | | | | | |
| | *Variant 1* | 864.17660233 | 923.17660233 | 676.91674894 | 0.8801029148 |
| | *Variant 2* | 1212.680211 | 1271.680211 | 1025.3158465 | 0.9232568649 |
| | *Variant 3* | 884.03996777 | 943.03996777 | 696.72697436 | 0.8484414876 |
| **6** | | | | | |
| | *Variant 1* | 905.63428619 | 976.63428619 | 680.28768296 | 0.8912028287 |
| | *Variant 2* | 1264.294607 | 1335.294607 | 1038.8222362 | 0.904546429 |
| | *Variant 3* | 929.24321804 | 1000.243218 | 703.83266664 | 0.8649570584 |
| **7** | | | | | |
| | *Variant 1* | 948.05853554 | 1031.0585355 | 684.62518247 | 0.8796664103 |
| | *Variant 2* | 1313.6175582 | 1396.6175582 | 1050.0371811 | 0.9115166283 |
| | *Variant 3* | 974.26163867 | 1057.2616387 | 710.75352929 | 0.8373914708 |
| **8** | | | | | |
| | *Variant 1* | 997.33433887 | 1092.3343389 | 695.81423595 | 0.8609592166 |
| | *Variant 2* | 1369.3686331 | 1464.3686331 | 1067.6802496 | 0.9224914632 |
| | *Variant 3* | 1026.4893892 | 1121.4893892 | 724.88372185 | 0.8434144154 |

Information criteria (smaller values indicate better fits, smallest values per LCA variant in bold). BIC = Bayesian information criterion, CAIC= Consistent Akaike information criterion, aBIC= Sample size adjusted BIC

Entropy = relative entropy (values close to 0 indicate poor class separation (i.e. random guessing), values close 1 indicate that classes are well-separated)

[a] In the three-class model, two classes are very similar to those in the four-class model. The third class seems to be a merged class of the two remaining classes in the four-class LCA.

[b] A five-class model maintained three of the four classes of the four-class model and subdivided a small part of the fourth class into a subgroup (prevalence below 3%). Class four did not essentially change.

## Appendix 6

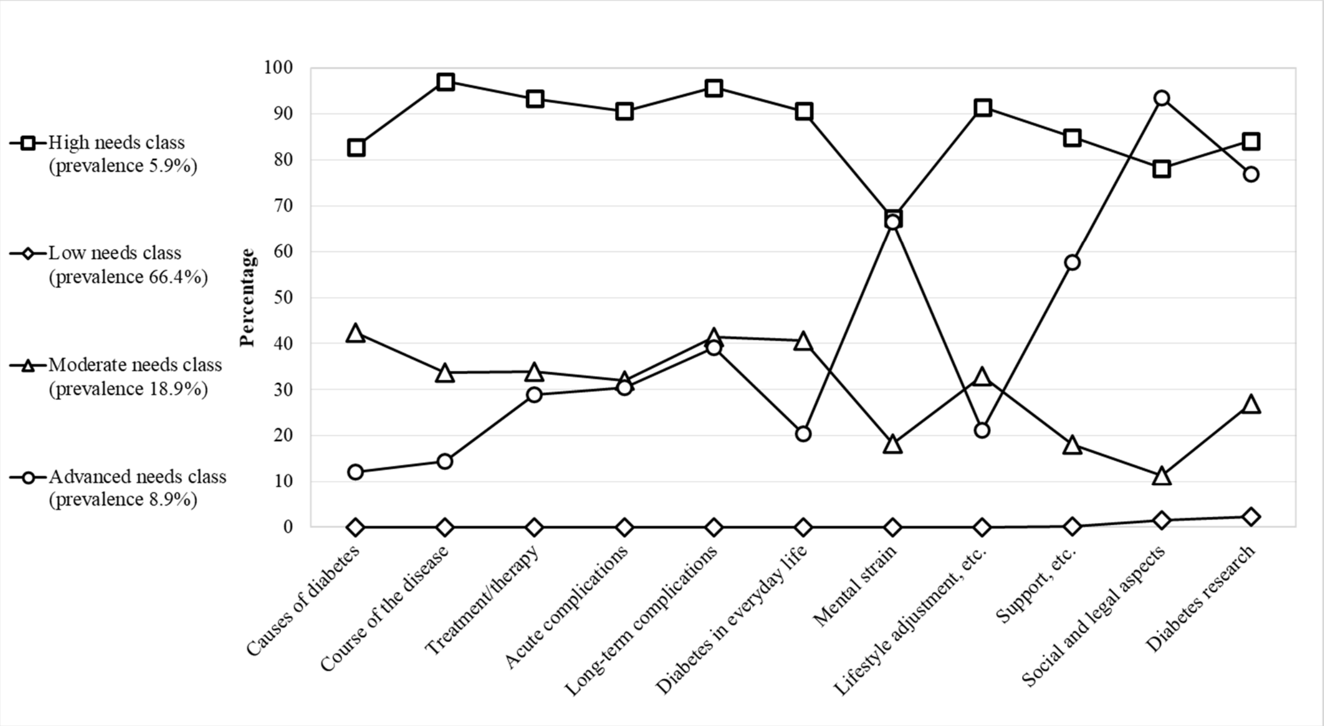


**Probabilities of information needs among people with diabetes stratified by the identified LCA classes in the sensitivity analysis (Variant 2) without covariates (n=837)**

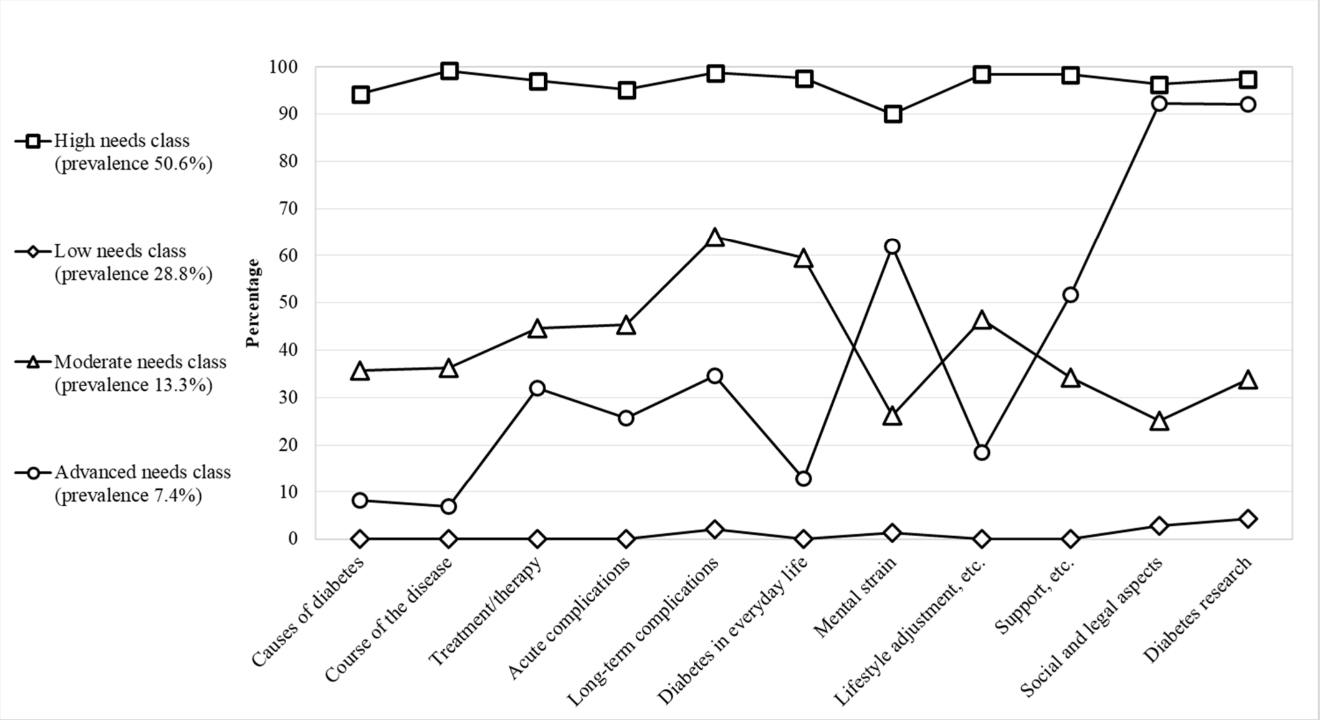


**Probabilities of information needs among people with diabetes stratified by the identified LCA classes in the sensitivity analysis (Variant 3) without covariates (n=613)**

# Appendix 7

*LCA with covariates per LCA variant (main analysis (Variant 1), n=306; sensitivity analysis (Variant 2), n=434; sensitivity analysis (Variant 3), n=390)*

| | | **Low needs vs. high needs** | | **Moderate needs vs. high needs** | | **Advanced needs vs. high needs** | | **Moderate needs vs. low needs** | | **Advanced needs vs. low needs** | | **Advanced needs vs. moderate needs** | |
|---|---|---|---|---|---|---|---|---|---|---|---|---|---|
| | | **OR** | **CI 95%** | **OR** | **CI 95%** | **OR** | **CI 95%** | **OR** | **CI 95%** | **OR** | **CI 95%** | **OR** | **CI 95%** |
| Age (years) | | | | | | | | | | | | | |
| | *LCA Variant 1* | **1.06** | **[1.03; 1.09]** | **1.04** | **[1.01; 1.07]** | 1.01 | [0.98; 1.05] | 0.98 | [0.95; 1.01] | **0.95** | **[0.92; 0.99]** | 0.97 | [0.94; 1.01] |
| | *LCA Variant 2* | **1.05** | **[1.02; 1.09]** | **1.04** | **[1.003; 1.08]** | 0.99 | [0.96; 1.03] | 0.99 | [0.96; 1.01] | **0.94** | **[0.92; 0.97]** | **0.96** | **[0.92; 0.99]** |
| | *LCA Variant 3* | **1.03** | **[1.01; 1.06]** | 1.01 | [0.99; 1.04] | 0.99 | [0.96; 1.02] | 0.98 | [0.95; 1.01] | **0.95** | **[0.92; 0.99]** | 0.97 | [0.94; 1.01] |
| Sex (female) | | | | | | | | | | | | | |
| | *LCA Variant 1* | 1.19 | [0.74; 1.91] | 1.22 | [0.71; 2.09] | 1.15 | [0.61; 2.17] | 1.03 | [0.62; 1.71] | 0.97 | [0.54; 1.75] | 0.94 | [0.49; 1.83] |
| | *LCA Variant 2* | 0.83 | [0.47; 1.45] | 0.99 | [0.54; 1.82] | 0.90 | [0.46; 1.76] | 1.19 | [0.81; 1.77] | 1.09 | [0.68; 1.74] | 0.91 | [0.52; 1.60] |
| | *LCA Variant 3* | 1.14 | [0.78; 1.68] | 1.10 | [0.70; 1.73] | 1.19 | [0.70; 2.03] | 0.96 | [0.60; 1.55] | 1.05 | [0.61; 1.81] | 1.09 | [0.59; 2.01] |
| Years of education (≥11 years) | | | | | | | | | | | | | |
| | *LCA Variant 1* | 1.01 | [0.63; 1.61] | 1.47 | [0.87; 2.48] | **2.21** | **[1.20; 4.09]** | 1.46 | [0.89; 2.39] | **2.19** | **[1.23; 3.91]** | 1.50 | [0.79; 2.86] |
| | *LCA Variant 2* | 0.72 | [0.42; 1.26] | 0.86 | [0.47; 1.57] | 0.93 | [0.48; 1.80] | 1.19 | [0.81; 1.75] | 1.28 | [0.80; 2.04] | 1.08 | [0.62; 1.88] |
| | *LCA Variant 3* | 0.98 | [0.67; 1.44] | 1.19 | [0.76; 1.85] | **1.97** | **[1.16; 3.33]** | 1.21 | [0.76; 1.94] | **2.01** | **[1.17; 3.48]** | 1.66 | [0.90; 3.05] |
| Type of diabetes (Type 2) | | | | | | | | | | | | | |
| | *LCA Variant 1* | **0.17** | **[0.05; 0.56]** | 0.74 | [0.12; 4.66] | 0.34 | [0.08; 1.38] | 4.34 | [0.89; 21.17] | 1.98 | [0.67; 5.83] | 0.45 | [0.07; 2.98] |
| | *LCA Variant 2* | **0.10** | **[0.01; 0.93]** | 0.28 | [0.03; 3.11] | 0.14 | [0.01; 1.52] | 2.88 | [0.97; 8.57] | 1.48 | [0.63; 3.47] | 0.51 | [0.14; 1.87] |
| | *LCA Variant 3* | **0.28** | **[0.12; 0.64]** | 1.67 | [0.32; 8.73] | 0.49 | [0.17; 1.39] | **6.08** | **[1.24. 29.70]** | 1.78 | [0.68; 4.68] | 0.29 | [0.05; 1.67] |
| Diabetes duration (years) | | | | | | | | | | | | | |
| | *LCA Variant 1* | **0.97** | **[0.95; 0.99]** | **0.97** | **[0.94; 0.998]** | 1.00 | [0.97; 1.03] | 1.00 | [0.97; 1.03] | **1.03** | **[1.001; 1.06]** | 1.03 | [0.995; 1.07] |
| | *LCA Variant 2* | 0.99 | [0.95; 1.02] | 0.98 | [0.94; 1.01] | 1.02 | [0.98; 1.06] | 0.99 | [0.97; 1.02] | **1.03** | **[1.01; 1.06]** | **1.04** | **[1.01; 1.08]** |
| | *LCA Variant 3* | **0.97** | **[0.95; 0.99]** | **0.97** | **[0.32; 0.99]** | 1.00 | [0.97; 1.02] | 1.00 | [0.97; 1.03] | **1.03** | **[0.9997; 1.06]** | 1.03 | [0.996; 1.07] |
| Antihyperglycaemic medication (yes) | | | | | | | | | | | | | |
| | *LCA Variant 1* | 0.88 | [0.44; 1.76] | 0.86 | [0.40; 1.85] | 0.77 | [0.30; 1.99] | 0.98 | [0.50; 1.90] | 0.87 | [0.37; 2.08] | 0.89 | [0.35; 2.26] |
| | *LCA Variant 2* | 0.70 | [0.30; 1.62] | 0.82 | [0.33; 2.03] | 0.80 | [0.28; 2.26] | 1.18 | [0.69; 2.01] | 1.14 | [0.54; 2.42] | 0.97 | [0.42; 2.25] |
| | *LCA Variant 3* | 0.91 | [0.53; 1.56] | 0.87 | [0.48; 1.57] | 0.76 | [0.35; 1.65] | 0.95 | [0.51; 1.76] | 0.84 | [0.38; 1.84] | 0.88 | [0.38; 2.04] |

| | Low needs vs. high needs | | Moderate needs vs. high needs | | Advanced needs vs. high needs | | Moderate needs vs. low needs | | Advanced needs vs. low needs | | Advanced needs vs. moderate needs | |
|---|---|---|---|---|---|---|---|---|---|---|---|---|
| | OR | CI 95% | OR | CI 95% | OR | CI 95% | OR | CI 95% | OR | CI 95% | OR | CI 95% |
| Comorbidities (yes) | | | | | | | | | | | | |
| *LCA Variant 1* | **0.44** | **[0.27; 0.73]** | **0.55** | **[0.31; 0.98]** | 0.83 | [0.43; 1.57] | 1.25 | [0.73; 2.12] | **1.86** | **[1.03; 3.38]** | 1.49 | [0.77; 2.92] |
| *LCA Variant 2* | **0.34** | **[0.19; 0.61]** | 0.56 | [0.30; 1.07] | 0.82 | [0.41; 1.63] | **1.66** | **[1.10; 2.50]** | **2.41** | **[1.49; 3.89]** | 1.45 | [0.82; 2.56] |
| *LCA Variant 3* | **0.62** | **[0.42; 0.92]** | 0.77 | [0.48; 1.23] | 1.20 | [0.71; 2.04] | 1.24 | [0.75; 2.04] | **1.94** | **[1.12; 3.37]** | 1.57 | [0.84; 2.90] |
| Current smoking behaviour (yes) | | | | | | | | | | | | |
| *LCA Variant 1* | 0.98 | [0.51; 1.89] | 0.44 | [0.18; 1.06] | **0.12** | **[0.03; 0.50]** | 0.44 | [0.19; 1.04] | **0.12** | **[0.03; 0.49]** | 0.28 | [0.06; 1.27] |
| *LCA Variant 2* | 0.64 | [0.29; 1.37] | 0.49 | [0.20; 1.21] | 0.40 | [0.15; 1.06] | 0.78 | [0.40; 1.49] | 0.62 | [0.29; 1.35] | 0.80 | [0.31; 2.07] |
| *LCA Variant 3* | 1.34 | [0.78; 2.32] | 0.45 | [0.20; 1.03] | **0.31** | **[0.10; 0.91]** | **0.34** | **[0.14; 0.79]** | **0.23** | **[0.08; 0.69]** | 0.68 | [0.18; 2.57] |
| High well-being (≥50) | | | | | | | | | | | | |
| *LCA Variant 1* | 1.21 | [0.71; 2.08] | 0.85 | [0.47; 1.56] | 0.67 | [0.34; 1.34] | 0.70 | [0.39; 1.26] | 0.56 | [0.29; 1.07] | 0.79 | [0.38; 1.63] |
| *LCA Variant 2* | 1.41 | [0.78; 2.55] | 1.62 | [0.85; 3.11] | 1.02 | [0.51; 2.05] | 1.15 | [0.73; 1.80] | 0.72 | [0.44; 1.20] | 0.63 | [0.34; 1.16] |
| *LCA Variant 3* | 1.34 | [0.87; 2.07] | 0.86 | [0.53; 1.40] | 0.92 | [0.52; 1.63] | 0.64 | [0.38; 1.09] | 0.69 | [0.38; 1.26] | 1.07 | [0.55; 2.07] |
| Diabetes education (yes) | | | | | | | | | | | | |
| *LCA Variant 1* | 1.13 | [0.70; 1.82] | 1.15 | [0.67; 1.98] | **1.98** | **[1.03; 3.82]** | 1.02 | [0.62; 1.69] | 1.75 | [0.94; 3.27] | 1.72 | [0.87; 3.39] |
| *LCA Variant 2* | 1.05 | [0.59; 1.87] | 0.97 | [0.52; 1.81] | **2.04** | **[1.004; 4.13]** | 0.92 | [0.62; 1.37] | **1.94** | **[1.15; 3.26]** | **2.10** | **[1.16; 3.80]** |
| *LCA Variant 3* | 0.94 | [0.63; 1.39] | 0.82 | [0.52; 1.28] | 1.51 | [0.86; 2.65] | 0.87 | [0.54; 1.40] | 1.61 | [0.90; 2.88] | **1.86** | **[0.99; 3.49]** |
| Current level of information | | | | | | | | | | | | |
| *LCA Variant 1* | **1.14** | **[1.09; 1.18]** | 1.01 | [0.97; 1.05] | **1.11** | **[1.05; 1.17]** | **0.89** | **[0.85; 0.92]** | 0.98 | [0.93; 1.03] | **1.10** | **[1.04; 1.16]** |
| *LCA Variant 2* | **1.18** | **[1.13; 1.24]** | **1.08** | **[1.02; 1.13]** | **1.14** | **[1.08; 1.21]** | **0.91** | **[0.88; 0.94]** | 0.97 | [0.93; 1.01] | **1.06** | **[1.01; 1.11]** |
| *LCA Variant 3* | **1.09** | **[1.06; 1.13]** | 0.99 | [0.95; 1.02] | **1.08** | **[1.03; 1.13]** | **0.90** | **[0.87; 0.94]** | 0.99 | [0.94; 1.03] | **1.09** | **[1.04; 1.15]** |
| Time preference (rather agree) | | | | | | | | | | | | |
| *LCA Variant 1* | **2.05** | **[1.24; 3.38]** | 1.18 | [0.66; 2.09] | 0.60 | [0.28; 1.27] | **0.58** | **[0.34; 0.97]** | **0.29** | **[0.14; 0.59]** | 0.51 | [0.24; 1.09] |
| *LCA Variant 2* | 1.54 | [0.85; 2.80] | 0.94 | [0.49; 1.81] | 0.64 | [0.30; 1.35] | **0.61** | **[0.41; 0.92]** | **0.42** | **[0.24; 0.71]** | 0.68 | [0.36; 1.27] |
| *LCA Variant 3* | **1.78** | **[1.20; 2.64]** | 1.20 | [0.75; 1.92] | 0.68 | [0.37; 1.27] | 0.67 | [0.42; 1.09] | **0.38** | **[0.21; 0.72]** | 0.57 | [0.29; 1.13] |

OR= odds ratio (corresponding to one unit change in age, diabetes duration and current level of information)
CI= confidence interval
**significant results (p<0.05)**

# Appendix 8

*Participants' characteristics stratified by classes per LCA variant with covariates*

| **Characteristics** | | **High needs class** n (%) / (M ± SD) | **Low needs class** n (%) / (M ± SD) | **Moderate needs class** n (%) / (M ± SD) | **Advanced needs class** n (%) / (M ± SD) |
|---|---|---|---|---|---|
| **N** | | | | | |
| | *LCA Variant 1* | 85 | 120 | 60 | 41 |
| | *LCA Variant 2* | 37 | 254 | 86 | 57 |
| | *LCA Variant 3* | 156 | 117 | 70 | 47 |
| **Age** | | | | | |
| | *LCA Variant 1* | 67.1 ± 9.1 | 70.3 ± 8.0 | 70.3 ± 9.5 | 65.9 ± 10.4 |
| | *LCA Variant 2* | 66.5 ± 9.8 | 70.5 ± 8.7 | 69.8 ± 9.5 | 65.9 ± 9.6 |
| | *LCA Variant 3* | 68.6 ± 9.1 | 70.1 ± 8.0 | 69.8 ± 10.0 | 66.0 ± 9.8 |
| **Female** | | | | | |
| | *LCA Variant 1* | 32 (37.6) | 55 (45.8) | 25 (41.7) | 18 (43.9) |
| | *LCA Variant 2* | 17 (45.9) | 100 (39.4) | 36 (41.9) | 21 (36.8) |
| | *LCA Variant 3* | 59 (37.8) | 52 (44.4) | 28 (40.0) | 22 (46.8) |
| **Years of education (≥11 years)** | *LCA Variant 1* | 34 (40.0) | 46 (38.3) | 30 (50.0) | 26 (63.4) |
| | *LCA Variant 2* | 19 (51.4) | 105 (41.3) | 40 (46.5) | 27 (47.4) |
| | *LCA Variant 3* | 65 (41.7) | 45 (38.5) | 33 (47.1) | 29 (61.7) |
| **Type of diabetes (Type 2)** | | | | | |
| | *LCA Variant 1* | 83 (97.6) | 109 (90.8) | 60 (100.0) | 35 (85.4) |
| | *LCA Variant 2* | 37 (100.0) | 235 (92.5) | 85 (98.8) | 50 (87.7) |
| | *LCA Variant 3* | 151 (96.8) | 107 (90.6) | 70 (100.0) | 41 (87.2) |
| **Diabetes duration (years)** | | | | | |
| | *LCA Variant 1* | 13.0 ± 10.5 | 11.9 ± 8.9 | 9.6 ± 9.6 | 14.9 ± 12.5 |
| | *LCA Variant 2* | 11.3 ± 10.6 | 12.4 ± 9.5 | 9.9 ± 7.4 | 17.4 ± 14.0 |
| | *LCA Variant 3* | 13.8 ± 10.6 | 11.7 ± 8.8 | 9.4 ± 9.1 | 14.1 ± 12.2 |
| **Antihyperglycaemic medication (yes)** | *LCA Variant 1* | 74 (87.1) | 101 (84.2) | 49 (81.7) | 37 (90.2) |
| | *LCA Variant 2* | 32 (86.5) | 214 (84.3) | 72 (83.7) | 53 (93.0) |
| | *LCA Variant 3* | 136 (87.2) | 98 (83.8) | 56 (80.0) | 42 (89.4) |
| **Comorbidities (yes)** | | | | | |
| | *LCA Variant 1* | 42 (49.4) | 40 (33.3) | 22 (36.7) | 24 (58.5) |
| | *LCA Variant 2* | 20 (54.1) | 86 (33.9) | 33 (38.4) | 35 (61.4) |
| | *LCA Variant 3* | 72 (46.2) | 39 (33.3) | 25 (35.7) | 25 (53.2) |
| **Current smoking behaviour (yes)** | *LCA Variant 1* | 15 (17.6) | 21 (17.5) | 4 (6.7) | 0 (0.0) |
| | *LCA Variant 2* | 7 (18.9) | 32 (12.6) | 7 (8.1) | 5 (8.8) |
| | *LCA Variant 3* | 21 (13.5) | 20 (17.1) | 3 (4.3) | 2 (4.3) |
| **High well-being (≥50)** | | | | | |
| | *LCA Variant 1* | 59 (69.4) | 93 (77.5) | 43 (71.7) | 28 (68.3) |
| | *LCA Variant 2* | 21 (56.8) | 193 (76.0) | 67 (77.9) | 37 (64.9) |
| | *LCA Variant 3* | 108 (69.2) | 91 (77.8) | 48 (68.6) | 34 (72.3) |
| **Diabetes education (yes)** | | | | | |
| | *LCA Variant 1* | 44 (51.8) | 68 (56.7) | 27 (45.0) | 32 (78.0) |
| | *LCA Variant 2* | 16 (43.2) | 138 (54.3) | 40 (46.5) | 45 (78.9) |
| | *LCA Variant 3* | 88 (56.4) | 65 (55.6) | 29 (41.4) | 36 (76.6) |
| **Current level of information** | *LCA Variant 1* | 13.0 ± 6.0 | 18.9 ± 7.0 | 11.8 ± 5.5 | 18.3 ± 4.3 |
| | *LCA Variant 2* | 10.7 ± 4.8 | 17.9 ± 6.6 | 13.4 ± 6.4 | 17.1 ± 4.7 |
| | *LCA Variant 3* | 14.7 ± 6.2 | 18.6 ± 6.9 | 12.5 ± 5.3 | 19.2 ± 4.9 |
| **Time preference (rather agree)** | *LCA Variant 1* | 23 (27.1) | 60 (50.0) | 21 (35.0) | 5 (12.2) |
| | *LCA Variant 2* | 12 (32.4) | 114 (44.9) | 26 (30.2) | 11 (19.3) |
| | *LCA Variant 3* | 51 (32.7) | 57 (48.7) | 25 (35.7) | 10 (21.3) |
